# Study of Subjective Data Integrity for Image Quality Data Sets with Consumer Camera Content

**DOI:** 10.3390/jimaging6030007

**Published:** 2020-02-25

**Authors:** Jakub Nawała, Margaret H. Pinson, Mikołaj Leszczuk, Lucjan Janowski

**Affiliations:** 1 AGH University of Science and Technology, MP 30059 Kraków, Poland; leszczuk@agh.edu.pl (M.L.); janowski@kt.agh.edu.pl (L.J.); 2 Institute for Telecommunication Sciences, National Telecommunications and Information Administration, Boulder, CO 80305, USA; MPinson@ntia.gov

**Keywords:** image quality, data integrity, consumer camera, blind quality assessment, evaluation, subjective data, no reference, NR metrics, subjective study

## Abstract

We need data sets of images and subjective scores to develop robust no reference (or blind) visual quality metrics for consumer applications. These applications have many uncontrolled variables because the camera creates the original media and the impairment simultaneously. We do not fully understand how this impacts the integrity of our subjective data. We put forward two new data sets of images from consumer cameras. The first data set, CCRIQ2, uses a strict experiment design, more suitable for camera performance evaluation. The second data set, VIME1, uses a loose experiment design that resembles the behavior of consumer photographers. We gather subjective scores through a subjective experiment with 24 participants using the Absolute Category Rating method. We make these two new data sets available royalty-free on the Consumer Digital Video Library. We also present their integrity analysis (proposing one new approach) and explore the possibility of combining CCRIQ2 with its legacy counterpart. We conclude that the loose experiment design yields unreliable data, despite adhering to international recommendations. This suggests that the classical subjective study design may not be suitable for studies using consumer content. Finally, we show that Hoßfeld–Schatz–Egger α failed to detect important differences between the two data sets.

## 1. Introduction

Consumer cameras are becoming more and more intelligent. Exciting possibilities await once cameras can evaluate visual quality problems “on the fly”. The camera could tell the user about problems and how to fix them—change the framing, avoid back-lit subjects, or move farther away (so the camera can focus adequately). The possibilities are virtually endless. All those functionalities require a set of technologies to be in place. Apart from object detection and scene understanding, a robust, No-Reference (NR) image and video quality metrics, designed to work with consumer cameras, are of key importance.

NR (or blind) visual quality metrics operate on pixel-level information (as decoded from a compressed image or an individual video frame). They objectively assess the subjectively perceived quality of visual materials. Due to its origins rooted in Quality of Service (QoS), this subjectively perceived quality relates to the quality of a stimulus after degradation (e.g., compression or lossy transmission). The naming convention reflects this property. Hence, “no-reference” corresponds to quality evaluation performed without any reference to the original, non-distorted material. This is in contrast to more classical Full-Reference (FR) algorithms. Those explicitly compare a distorted visual footage to its unaltered version. Although the research in the domain of NR image quality assessment (IQA) is still active, there exist well-established solutions. One such example is the BRISQUE model [1] with its characteristic property of using natural scene statistic-based features. This allows it to operate in a distortion-generic fashion. This is in contrast to other solutions that provide distortion-specific quality indication [2]. Importantly, one of the newest trends is to use the existing neural network architectures (dedicated for object detection) and, building upon them, propose better performing NR IQA solutions [3].

Our purpose is to encourage and advance the research of quality evaluation for user-generated content. We are specifically focused on the development of NR IQA algorithms. An automated method of assessing consumer content may prove significant for a multitude of applications. Apart from the already mentioned photographer assistance (requests to change the framing or lighting conditions), NR metrics could serve more general purposes. If considered as aesthetic evaluators, they could predict how people would judge a given content. This could help build more attractive multimedia services. NR metrics could also suggest which images a user may want to delete first from their device (to free up storage). Similarly, they could recommend a potentially best looking profile picture or wallpaper.

In the internal computational pipeline of NR metrics, digital image processing extracts meaningful information. Still, this and related techniques alone are not enough. Robust subjective data sets are crucial to train and verify NR metrics. These data sets must reflect the myriad of scenes, lighting conditions, and camera capture problems that users encounter. Only by doing so can they claim to be genuinely referenceless. The ideal NR metric should be able to judge the quality of any content, including user-generated content. It should respond gracefully to all possible content varieties: outdoor and indoor shots, panoramas and close-ups, static and dynamic scenes, as well as those of low and high dynamic range.

Unfortunately, most of the freely available subjective image and video quality data sets only analyze coding, network, and artificially introduced impairments [4,5,6,7,8,9]. This is the classical approach, where original material (also referred to as Source Reference Circuit, SRC) is processed through a set of degradation mechanisms (conventionally called Hypothetical Reference Circuits, HRCs) to produce the test materials of interest (also called Processed Video Sequences, PVSs). This approach has several consequences. First, the HRCs span mainly transcoding and transmission impairments (e.g., different compression algorithms and various bit-rates, packet loss ratios, resolution reductions, and format conversions). It is hard to imagine that this set exhaustively represents distortions encountered by users of consumer cameras. Second, analogue noise is rare and is usually only artificially introduced to photos (although it can be found in consumer images). Third, the source (reference) images and videos (SRCs) are restricted to “good quality or better.” This is not in line with typical consumer conditions. There, we expect algorithms to respond gracefully to low-quality visual input. They stand no chance of providing this functionality if tested only on good quality visual stimuli.

Additionally, since subjective data sets contain scores gathered in subjective assessments (also called subjective experiments) [10], the subjective experiment design influences the content of the data sets. Each experiment has a limited number of SRCs and HRCs. Camera impairments are a confounding factor that would complicate the analysis of the results. Thus, most freely available data sets intentionally exclude SRCs with flawed camera capture. Furthermore, the extensive use of the Absolute Category Rating with Hidden Reference (ACR-HR) test method [11,12] further intensifies this phenomenon. This test method explicitly prohibits SRCs that are fair, poor, or bad quality.

All of the typical data sets described are ill suited for consumer content evaluation. For user-generated content, impairments come from the camera capture pipeline (e.g., lens, sensors, image processing, encoder, decoder, and display) and human factors (e.g., the skill of the photographer, framing, and subject matter aesthetics). Codec impairments are typically minimal due to adequately high bit-rates. The only way to exercise other variables (not related to the codec) is to create a large variety of photos and videos; the impairments from consumer cameras cannot be simulated with software. These requirements are challenging, so there are few data sets available to train NR IQA metrics for consumer content evaluation.

A number of existing data sets do contain a sufficiently broad scope to provide training data for NR metrics aimed at evaluating consumer content. The “CID2013 Camera Image Database” [13] contains photographs from a variety of consumer cameras (DSLRs, compacts, and phones). Thus, there is a greater possibility that this database offers a good representation of consumer camera impairments. “ITS4S” [14], “ITS4S2” [15] and “ITS4S3” [16] data sets present a series of image and video quality experiments designed specifically to provide training data for NR metrics. Thus, they mostly contain material with distortions stemming purely from the capturing process. Furthermore, they emphasize a large variety of unrepeated content. This means that there are only a few multimedia materials that are repeated in the same session of a subjective evaluation test. (The three data sets can be found on the Consumer Digital Video Library (CDVL, www.cdvl.org) [17] by searching for the keywords “its4s”, “its4s2” and ”its4s3”, respectively.) Next is the “LIVE In the Wild Image Quality Challenge Database” [18]. It includes 1162 unique photographs from mobile devices. Containing a huge variety of subject matter and camera impairments, it also fits the consumer-related scope well. There is also its video counterpart called “LIVE-Qualcomm Mobile In-Capture Video Quality Database” [19,20]. It contains 208 videos captured using eight mobile devices. The database content models six common capture related distortion categories. Importantly, each video is paired with subjective assessments of 39 subjects. To the best of our knowledge, the largest existing data set of authentically distorted images is the “KonIQ-10k Image Database” [21]. It contains 10,073 unique photographs, each scored by 120 crowd workers. The KonIQ-10k images are a subset of an even larger, diverse data set of 100 million multimedia objects [22]. The authors of [21] use a specially developed technique to sample the larger data set properly. This, combined with the data set size, highlights its value for NR metrics development. The same team has also prepared the “KoNViD-1k Video Database” [23,24]. Utilizing a similar processing pipeline, it contains 1200 natural, real-world videos accompanied by subjective scores of crowd workers. Finally, the “Consumer-Content Resolution and Image Quality” (CCRIQ) data set [25] focuses on consumer content evaluation by design.

The key idea of this paper is to encourage NR IQA algorithms development for consumer content. To this end, we address three aims. The first one is to provide subjectively evaluated consumer content. Thus, we put forward two new data sets. Use of those data sets should allow better NR metrics to be built. In particular, we expect them to widen the scope of NR metrics and improve their predictive abilities. The second aim is to explore the applicability of the classical subjective study design for evaluating the quality of consumer content. It seems logical to apply the classical approach to this new stimulus type. We check whether proceeding this way produces consistent results. This also leads to our last aim. We want to provide guidelines on subjective data integrity analysis. For this, we use two classical approaches and propose one non-standard method.

When designing our subjective data sets, we hypothesize that images that better reflect consumer camera usage can be used in the classical subjective experiment setup (hypothesis #1). Specifically, we use post-processed images, as well as a mix of vertical and horizontal shots in a single test session. These two choices diverge from recommended practices. We also theorize the Hoßfeld–Schatz–Egger (HSE) α coefficient [26] can be used to assess whether our results are similar to typical image quality subjective studies (hypothesis #2). We expect such a similarity to indicate results integrity. (In the original paper, the parameter is called *SOS*. We call it *HSE* (from Hoßfeld, Schatz, and Egger) because of SOS being traditionally used to denote standard deviation of opinion scores.)

Two main conclusions result from our work. First, we present a case where the classical subjective study design ceases to work. This finding sheds some light on a trade-off between experiment repeatability and resemblance of a laboratory design to actual conditions of interest (i.e., its ecological validity). Second, we show that promising HSE α should not be used as an ultimate measure of the similarity of data sets. Two studies may report similar HSE α parameters, where, in reality, one presents consistent, and the other, inconsistent results.

As you read, remember that our goal is training data for NR metrics. The subjective experiment designs presented in this paper would not be appropriate for comparing and contrasting the performance of different cameras. To this end, a more methodical approach would be required. Specifically, dedicated test charts and a strictly controlled environment would be necessary. For more details, please refer to the IEEE 1858-2016 standard [27].

The remainder of this paper is structured as follows. In Section 2, we present the new CCRIQ2 and VIME1 data sets. With Section 3, we put forward our results. Specifically, Section 3.1 provides a high-level review of the data sets, Section 3.2 verifies their integrity, and Section 3.3 explores the possibility of combining CCRIQ2 with its legacy counterpart. Finally, materials and methods we use are described in Section 4. Our conclusions in Section 5 close the paper.

## 2. New Data Sets

With this work, we present two IQA data sets: CCRIQ2 and VIME1. The two contain scores from two subjective tests (subjective assessment) sessions. The first session, CCRIQ2, expands the CCRIQ data set. The second session, “Video and Image Models for Consumer Content Evaluation Data-set One” (VIME1), is entirely new. The combined data set, CCRIQ2 + VIME1, is freely available for research and development purposes and royalty-free to download from CDVL [17] (search for keyword “ccriq2+vime1”). See the CDVL [17] for licensing terms. We also make the subjective scores (without the visual content) available as a Supplementary Materials of the paper. (See the Supplementary Materials section at the end for a description of the data format and a download link.) Table 1 comprehensively compares our data sets with those already existing. The following sections further describe the two in more detail.

### 2.1. CCRIQ and CCRIQ2

We start by describing the legacy, CCRIQ data set. Its description helps to explain the design choices of the newer, CCRIQ2 data set introduced with this paper. The CCRIQ data set [25] contains the same 18 scenes, photographed with 23 digital cameras. The cameras range from 1 to 20 megapixels (MP) and include an approximately equal distribution of phones, tablets, point-and-shoots, and higher-end digital single-lens reflex (DSLR) cameras. CCRIQ evaluates consumer cameras, so amateur photographers held each camera (no tripods) and used automatic settings. All photos of a single scene were taken by a single photographer, who tried to achieve uniformity of lighting, composition, and framing. The images were subjectively rated on the Absolute Category Rating (ACR) scale at three labs: Intel, National Telecommunications and Information Administration’s Institute for Telecommunication Sciences (NTIA/ITS), and the University of Ghent. The images and ratings are available on the CDVL [17]. The 18 scenes selected for the CCRIQ data set were drawn from a broader set of 69 scenes. The choice of which scenes to retain was difficult, with many of the remaining 51 scenes having appealing content and desirable characteristics.

**Figure 1:**
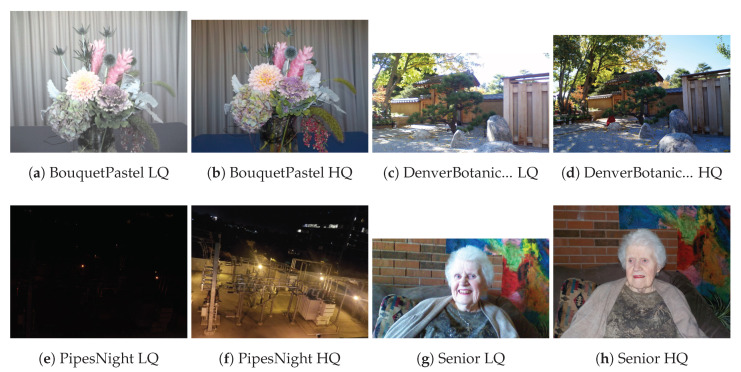
Sample of the CCRIQ2 scenes with Low Quality (LQ) and High Quality (HQ).

The new CCRIQ2 data set contains 88 photographs that depict four (4) scenes that were excluded from the original CCRIQ data set due to size constraints. They are captured with the same set of 23 cameras and also come from the larger set of 69 scenes. However, there is no overlap with the scenes used in the original CCRIQ data set. Importantly, this data set not only uses the same cameras as CCRIQ, but also retains the same image capture methodology (for more details, see [25]). Figure 1 presents the four scenes. We show a pair of sample images for each scene. One image from the pair represents a low quality sample (the LQ label) and the other a high quality sample (the HQ label). We have chosen the image samples manually with the intention of showing the quality spectrum of this data set. Only four scenes are used to not make the subjective study too long. This is a concern since each one new scene translates into 23 new images for which a number of subjective scores has to be gathered. The next paragraph details why we chose to use those specific four scenes.

The *Bouquet Pastel* scene depicts a pastel flower arrangement on a blue table in front of a cream curtain. The flowers were ≈0.5 m to 0.8 m from the camera, and the curtain was 1.5 m behind the flowers. The camera faces away from a wide bank of NE facing windows with bright diffuse light (35 lx). *Bouquet Pastel* shows interesting differences among the 23 cameras that may not be obvious from CCRIQ’s 18 scenes. *Denver Botanic Gardens Rocks*, photographed in the early afternoon at the Denver Botanic Gardens, depicts fine-textured gravel and, on occasion, lens flare. The camera was in dappled shade on a sunny day with no clouds. The lighting at the camera was ≈2000 lx; the lighting in the sun was ≈10,000 lx. *Pipes Night* shows an interesting night composition mostly devoid of vertical and horizontal lines. *Pipes Night* demonstrates camera response to the challenges of night photography. *Senior* depicted an older woman in front of a brick wall and was chosen to evaluate the camera’s response when depicting people (e.g., skin tones, shading of the face). *Senior* has a mix of dim natural lighting (from the left) and camera flash. Light measurements are not available for these two compositions.

The CCRIQ2 camera names (*A* through *X*) are identical to those used in the CCRIQ data set. An important difference between the data sets is the resolution of monitors used to conduct subjective studies. Where CCRIQ used High-Definition (1920 × 1080) and 4K (3840 × 2160) monitors, CCRIQ2 used (1440 × 900). Importantly, CCRIQ2’s testing software imposed a limitation on image resolution. All images were presented re-scaled to 720 pixels in height.

### 2.2. VIME1

Video Quality Experts Group (VQEG) formed the VIME group [28] to investigate NR perceptual quality evaluation for consumer cameras and user-generated content. VIME focused on collaboration and information sharing with regard to NR image and video quality assessment for consumer devices. With the significant changes over the years in how consumers are capturing, manipulating, and sharing images and videos, VQEG felt it was time to develop assessment tools for such scenarios. VIME builds on the years of expertise that VQEG has around subjective and objective methodologies. VIME was recently replaced with the No-Reference Metric (NORM) group, which has a broader scope.

VIME organized events to capture photos of the same (or very similar) scenes, using various consumer cameras. The *VIME Image Database*, stored at Flickr and currently containing 670 images, is designed for developing and testing image models for consumer content evaluation [29]. The *VIME Image Database* contains photographs contributed by interested people, plus photographs taken during events organized at VQEG meetings. Attendees brought their cameras and went as a group to photograph the same scenes with different cameras.

**Figure 2:**
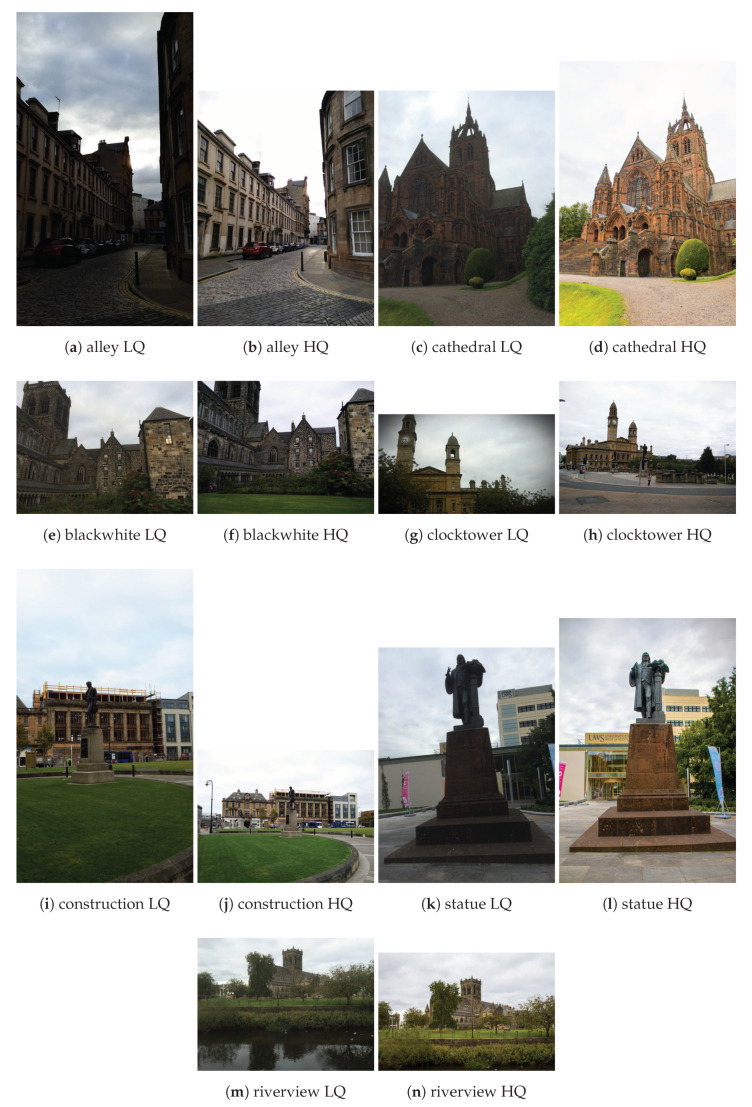
Samples of the VIME1 scenes with Low Quality (LQ) and High Quality (HQ).

The new VIME1 data set contains a selection of 101 photos from the *VIME Image Database*. Photos are limited to the same scene captured by 11 cameras. Only a few of the images from the *VIME Image Database* satisfy this requirement. Having such a set, it is possible to at least roughly compare the cameras amongst each other (although this would not replace a more methodical comparison [27]). This also resembles the CCRIQ2 design, as each camera performance is exercised by taking images in various conditions. However, this time there are many photographers taking shots simultaneously and hence the data set can be created faster. In other words, more test stimuli can be created in a shorter time. All the selected photographs depict cityscapes taken in the early evening in Glasgow, Scotland, during the September 2015 VQEG meeting. This makes the images thematically consistent. Again, this is uncommon among the photos from the larger database, where most images are one of a kind. VIME1 contains seven scenes, which are shown in Figure 2. As previously, we show both low quality (the LQ label) and high quality (the HQ label) image pairs for each scene. Since, unlike the CCRIQ2 data set, each scene was photographed by multiple photographers, VIME1 contains more significant variations in composition, framing, and camera location. The data set design is not a full matrix, as some people did not attend the entire event. (*Full matrix* means here each location photographed by all photographers. Since the VIME1 data set does not include images of all locations taken by all photographers, it is not a full matrix design.)

## 3. Results

We now continue with a more in-depth analysis of the combined data set. We examine the two individual data sets by applying a two-step procedure. First, we perform a high-level review of the data. This includes considering its positive and negative features, as well as comparison with similar, legacy data sets. Second, we verify data integrity. Here, we apply an informal overview of a set of scatter plots and two more formal methods. Those include a post-experimental screening of subjects (as recommended in Recommendation ITU-T P.913 [30]) and HSE α parameter analysis [26].

### 3.1. Experiment Design Review

The following subsection presents our subjective review of CCRIQ2 and VIME1 data sets. We go through their properties, underlining those especially important for NR metrics. We also highlight the differences between the two and existing data sets. This provides insight into the new contributions those data sets bring to the field of NR metrics development.

#### 3.1.1. CCRIQ2

We start by considering the positive features of CCRIQ2. Its primary advantage is the use of realistic impairments. All distortions come entirely from properties of a given camera. It guarantees the quality range does not have to be manually selected. It is naturally generated by responses of the cameras used. Taking into account the broad spectrum of resolutions, contents, and devices utilized, this quality range appears sensible. Especially important are differences in color reproduction between the devices. Following the authors of [31], we expect *color naturalness* to be one of the most important factors influencing the quality judgement. Having many photographs of the same scene, each with differently reproduced colors, further assures that we well explore the quality range.

Another positive side of this data set is its applicability to train and test NR metrics. Although many artifacts are superimposed in every single image, it is the overall quality that is interesting (and is captured in the subjective test). Furthermore, the appearance of artifacts is only loosely controllable or not controllable at all. In addition, the lack of a reference image to compare to makes the usage scenario of this data set clear. Only NR models for image quality assessment may be trained with this data. We consider this a positive feature since there are not many data sets strictly dedicated to those models. The existence of such data sets is crucial to sustaining NR models’ development.

When it comes to disadvantages of the CCRIQ2 data set, the superposition of multiple distortions (artifacts) is one crucial example. Dynamically changing lighting conditions, different shot angles, and the different internal processing of device all add up together and appear in the final image. What makes this problem even more severe is that all of those individual artifacts are difficult or impossible to control. This hinders data set applicability for performing artifact-specific analyses.

Another shortcoming of CCRIQ2 is its limited scope. This is because only four scenes are represented. Thus, although applicable, this data set alone is probably not sufficient to train and test a full-fledged NR metric. One possible mitigation is to combine this data set with its legacy counterpart, namely CCRIQ. We explore this idea in Section 3.3.

A comparison of CCRIQ2 with legacy data sets highlights a few critical differences. The first one is a redefinition of the SRC and HRC. Here, the SRC represents a scene and the HRC is a mixture of factors like device used, lighting conditions, and position of dynamic objects. This mixture comes from scenes not being static. Lighting conditions change from one image to another. The same is true for objects and people present on the scene. It contradicts a traditional design where most factors are controlled. Nevertheless, this dynamic nature of the data set is in line with conditions specific to the consumer scenario. Another significant difference is the amount of time necessary to create such a data set. One must take multiple shots of the same scene, each time changing the camera. The process is much more time consuming than an automatic injection of distortions (widespread in standard data sets). Although there are many differences, there is one property that makes CCRIQ2 similar to legacy data sets. It is the precise selection of cameras, their properties, and scenes captured. All span a range wide enough to provide meaningful results. This careful selection resembles standard data sets; most influencing variables are strictly controlled.

#### 3.1.2. VIME1

Following are general thoughts about the second data set, namely VIME1. Similarly to CCRIQ2, it represents realistic, consumer-scenario related impairments. The range of those is also defined purely by the limitations of the cameras used. Therefore, it is perceived as a good source of data for training and testing of NR image quality assessment models. A distinct and essential property of VIME1 is its consideration of selected post-processing operations. Some captured scenes appear in different versions, each representing a different post-processing operation (contrast adjustments or framing). This approach addresses the not so well-researched area of image aesthetics.

Inevitably, the VIME1 data set has some disadvantages. The parallel existence of many impairments makes it more difficult (or impossible) to identify the main factor influencing the quality. In addition, the severity of most of the impairments is not controlled. A narrow range of contents (mostly buildings and statues) and a small spectrum of resolutions are two scope-limiting factors. In addition, different scenes are captured by different devices. This impedes device-to-device comparisons. Another disadvantageous source of variability is the lack of a single photographer. In other words, the same scene is captured by many people. Finally, there are both portrait and landscape orientation shots. Taking into account that our subjective software rescales all images to be 720 pixels in height, vertical images may seem to be of worse quality (even when it is not the case).

Let us now compare VIME1 with similar legacy data sets. Due to its intrinsic coherence with CCRIQ2, VIME1 also uses a different notion of SRC and HRC: the first represents a scene and the second a mixture of factors related to a hand-held shot. The lack of full control over quality degrading factors is not in line with legacy data sets designs (and also resembles CCRIQ2). Furthermore, the creation of a data set like VIME1 takes a lot of time. Nevertheless, its loose restrictions on content and devices used make this process faster than in the case of CCRIQ2. Among all of the differences, the most important one is the use of post-processing. In particular, VIME1 contains images with post-processing aimed at improving the aesthetic properties of an image. This includes operations such as: adding a frame, applying the sepia filter, performing color and shadow correction, vignetting, or adapting contrast and exposure. The post-processing makes VIME1 particularly useful and valid for consumer-related scenarios.

A comparison of VIME1 and CCRIQ2 leads to a couple of observations. VIME1 uses convenience sampling to select cameras; CCRIQ2 uses a strict camera selection procedure to choose devices spanning a wide range of classes (smart-phones, tablets, point-and-shoots, and DSLRs), resolutions (from 1 to 20 MPs), and production dates (from 2000 to 2014). VIME1’s loose experiment design can be rapidly implemented with the aid of several assistants using their own devices; CCRIQ2’s strict design requires a regimented procedure of camera selection, subject matter specification, and photography technique. VIME1 contains more scenes, but the camera association is haphazard (e.g., three photographs from one camera and none from another); CCRIQ2 has fewer scenes but systematic photography (i.e., a full matrix of scenes and cameras, with only a few missing photographs). Counter-intuitively, the broader set of scenes in VIME1 does not correspond to a broader range of subject matter and impairments. VIME1 contains images of buildings and statues but lacks everything else (e.g., people, landscapes, building interiors, night scenes). CCRIQ2 actually has a broader range of subject matter and impairments because each scene was carefully selected. One significant upside of VIME1 is its use of post-processing, which CCRIQ2 omits. Basically, VIME1 realistically reproduces real-life consumer conditions, while CCRIQ2 systematically evaluates camera performance.

### 3.2. Data Integrity Check

Having described our subjective assessment of the new data sets, we now follow with an objective evaluation. We start with the subjective scores from the CCRIQ2 data set. Then, we present a corresponding analysis of VIME1.

#### 3.2.1. CCRIQ2

**Figure 3:**
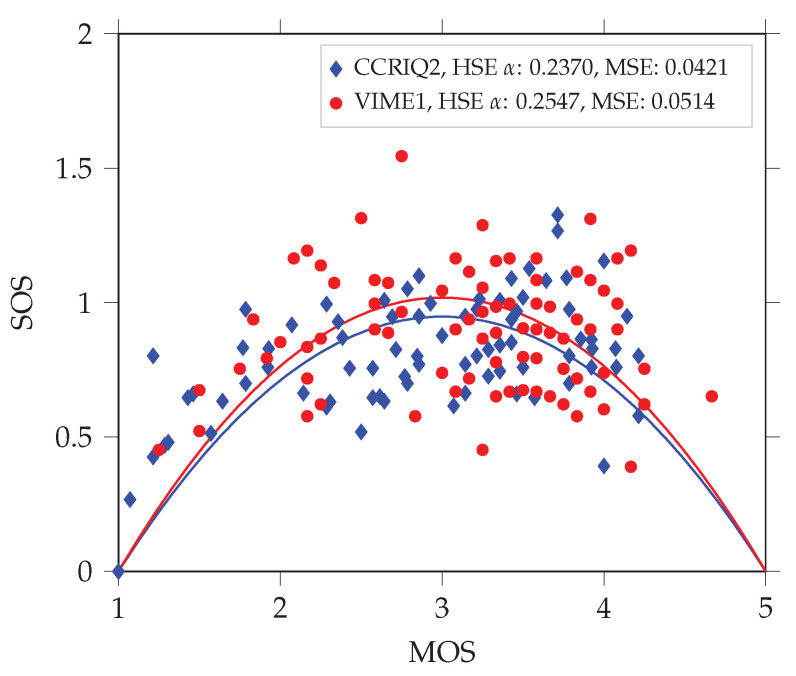
A comparison of HSE α parameters between the VIME1 and CCRIQ2 data sets. We use the α parameters to fit the quadratic function proposed in [26]. It models the dependency between mean opinion scores (MOS) and standard deviation of opinion scores (SOS). We analyze both data sets without unreliable subjects (screened according to [30] with the threshold correlation set to 0.6).

Following [26], we expect the data set to have an HSE α parameter in the range of 0.01 to 0.22. However, this is not the case. It has a value of 0.2370 instead. Figure 3 presents a corresponding quadratic function fit (with mean squared error (MSE) of 0.0421). This value suggests a level of variability larger than in traditional image quality assessments. In particular, it makes the data set similar to crowd-sourced subjective studies—those being known for higher score variability. The probable reason is the consumer-oriented nature of the data set. It introduces several factors related to larger variability. Section 3.1 lists and details each of those.

**Figure 4:**
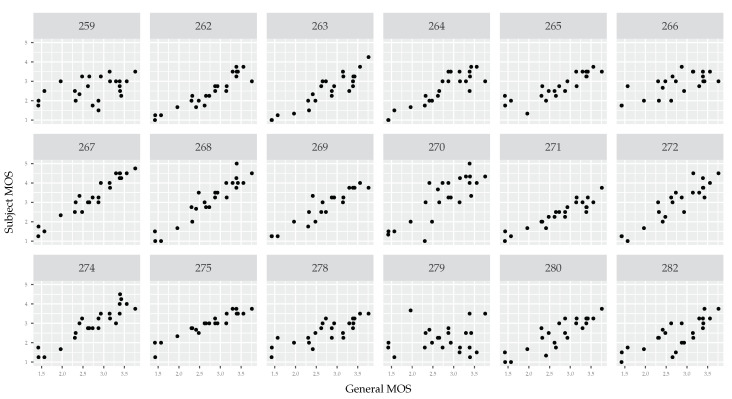
Scatter plots of each subject’s MOS (vertical axis) compared with the MOS of all subjects (horizontal axis). The data comes from the CCRIQ2 data set. We calculate per-subject MOS scores by aggregating individual scores by camera types. In general, opinion scores are consistent and correlated. There are few outliers like subjects number 259 and 279. We do not show a scatter plot for tester 283. This way, it is easier to compare this plot with the one in Figure 5. Due to the same testers being included in both data sets, there is a one-to-one scatter plot correspondence between the two figures.

Scatter plots comparing each subject’s opinion with the opinion of all subjects (see Figure 4) show a sufficient level of consistency. A few subjects deviate from the general opinion, but they constitute only a small fraction of all subjects.

The application of post-experimental screening of subjects with a threshold for minimal correlation of 0.75 (a value recommended in [30] for subjective testing of entertainment videos with opinion scores expressed on the 5-level categorical scale. We use this value first since our subjective experiment is using the same scale) discards 8 (out of 19) subjects. Looking at Figure 4 (with generally consistent opinions) and considering high HSE α of this data set, we can suspect that this screening is too conservative. Hence, we lower the threshold correlation to 0.6. It results in five discarded subjects.

Overall, we conclude that this data set is sufficiently consistent. Its slightly higher variability most probably stems from its consumer-oriented nature. However, we underline that the 14 subjects, who pass the post-experimental screening do not constitute a large sample of subjects population. We thus advise treating those subjective scores with a grain of salt.

#### 3.2.2. VIME1

As previously, we start with a consideration of the HSE α parameter. This time, its value is even higher and equals 0.2547. Figure 3 shows the corresponding quadratic fit (with MSE of 0.0514).

**Figure 5:**
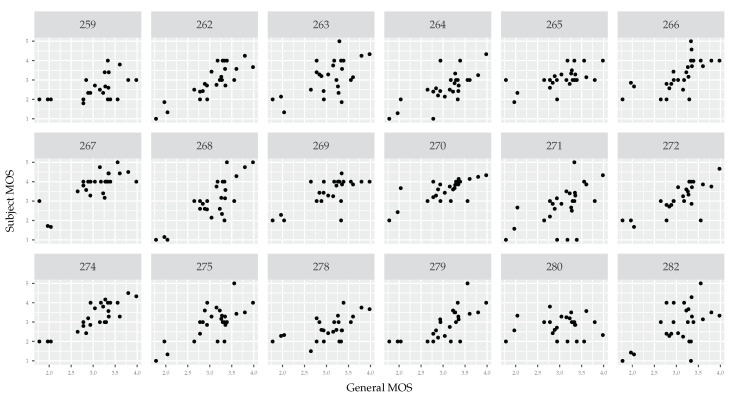
Scatter plots of each subject’s MOS (vertical axis) compared with the MOS of all subjects (horizontal axis). The data comes from the VIME1 data set. We calculate per-subject MOS scores by aggregating individual scores by camera types. There is almost no consistency in opinion scores. Only a few subjects are close to the general opinion. Among those are subjects 262 and 274. We do not show scatter plots for testers 273, 276, and 281. This way, it is easier to compare this plot with the one in Figure 4. Due to the same testers being included in both data sets, there is a one-to-one scatter plot correspondence between the two figures.

Although the HSE α parameter does not seem outright wrong, scatter plots draw a completely different picture (see Figure 5). There is almost no consistency in opinion scores. Where the scattering of subject opinions in Figure 4 shows that each subject generally agrees with other subjects about the relative ratings (i.e., a tight scatter of noise around a line), the scattering of subject opinions in Figure 5 shows large differences of opinion (see for example subjects 263, 271, 275, and 280). It is especially interesting if we consider that the same pool of testers is utilized in both data sets. Although fatigue seems to be a potential explanation, we must rule it out. The VIME1 images were the first ones presented to all testers.

The data inconsistency is further highlighted when we apply the screening. Using a threshold correlation of 0.75 discards 17 subjects (out of 21), and lowering the threshold to 0.6 filters out nine subjects (almost half of the total pool).

There are a few potential explanations for the inconsistency. First, to the best of our knowledge, this is the first subjective test with this design. Therefore, we can treat it as an exploratory, pilot study. Our choice of design comes from its similarity to the real-life consumer behaviors. The inconsistency of the results draws a line marking the limitation of this design. When adapting the classical approach to subjective testing, this design moves too far away from its area of applicability. Apart from having an exploratory nature, we theorize that the design has properties that can be considered flawed. It contains too few scenes and mixes vertical and horizontal shots.

Furthermore, different shots of the same scene come from different photographers. This results in multiple compositions in shots of the same scene. Finally, the pool of scenes represents a convenience sampling (and not a well-controlled choice). The photographs contain scenes of places that were easy to visit and capture for a group of people over about two hours.

We conclude that the subjective data from this data set is undoubtedly inconsistent. We do not recommend using it for training NR metrics. However, the images alone are a useful sample of photographs captured in real-life conditions. They also contain consumer-type post-processing, which is rare to find in existing data sets. One additional property of this data is that it can be used to explore potential explanations for its inconsistency. Maybe there is a way to analyze this data set in a way that produces inter-rater correlated results. We invite the community to use it for this and similar investigations.

#### 3.2.3. HSE α Reliability

As we show in Section 3.2.2, the HSE α parameter may misleadingly indicate results integrity. We theorize this is the case since according to its authors the parameter both tests for subjective results reliability and shows whether two subjective studies can be directly compared (or even pooled). If we follow these suggestions and take a look at VIME1’s HSE α (0.2547), we could conclude that VIME1 contains consistent results. This conclusion could be justified for at least three reasons. First, the HSE α value does not exceed the range of values reported in [26] for various types of subjective studies (0.0377–0.5902). Second, the corresponding MSE (0.0514) is also in the interval of values reported in [26] (0.0047–0.2204). Third, even if we compare VIME1’s HSE α to those of classical image and video streaming subjective studies (approx. 0.01–0.22; also taken from [26]), the slightly higher value can be attributed to the utilization of natural consumer device generated distortions (and not those artificially introduced).

With Section 3.2.2, we hope to highlight that the seeming consistency of VIME1 (as indicated by its HSE α) does not represent the actual state. To see the full picture, it is necessary to calculate other data integrity indicators as well. Two such examples are the post-experimental screening of subjects (as defined in Recommendation ITU-T P.913 [30]) and our suggested method of comparing each subject’s opinion with the opinion of all subjects (see Figure 4 and Figure 5). Thus, we do not recommend using the HSE α parameter alone to decide whether subjective data are consistent. Similarly, we think it would be unreasonable to pool multiple subjective data sets based just on their HSE α parameters having similar values.

Although our results suggest that the HSE α parameter is not a reliable tool for comparing multiple data sets, it would be unfair not to acknowledge its added value when compared to solely using MOS scores. It captures an important notion of scores variability that is completely ignored by MOS. We also point out that the HSE α parameter authors do not test in their work [26] how it responds to consumer content data sets like ours. Though not very probable, it may suggest that consumer content data sets fall outside the scope of HSE α applicability.

### 3.3. Comparison of CCRIQ and CCRIQ2

We now present a statistical comparison between subjective scores given to images from the CCRIQ and CCRIQ2 data sets. Those two are compared as they test the same set of 23 cameras. Although the same cameras are used, different scenes are captured. This means there is no shared content between CCRIQ2 and CCRIQ. Such a situation contradicts the classical design of subjective tests, whose results are intended to be combined. Two reasons justify our approach. First, CCRIQ and CCRIQ2 data sets are aimed at testing NR metrics. All classical designs do not consider this goal so other practices may be of interest. One of them is to not provide any content that is shared between tests. The second reason is the architecture of CCRIQ and CCRIQ2 data sets. Adding shared content would require amending CCRIQ2 with 23 images already tested in CCRIQ. Naturally, this would eliminate other not yet tested images and reduce the scope of the test.

Since subjective scores come from two different tests, there are further natural differences. In particular, there are different tester groups and different testing environments. The latter forced us to narrow the comparison. We only analyze a subsection of CCRIQ scores. To be more precise, CCRIQ2 images are presented during our subjective study in a resolution much smaller than 4K. Not wanting to make the resolution gap the most probable reason for differences in scores, we only consider scores for the High-Definition Monitor from CCRIQ. Importantly, in CCRIQ2, images are presented in a resolution not only smaller than 4K but also smaller than High-Definition. (During the subjective study, all images are scaled to have a height of 720 pixels.) This allows us to expect similarity in scores for low-resolution images. On the other hand, we expect high-resolution images to show significant differences between CCRIQ and CCRIQ2.

**Figure 6:**
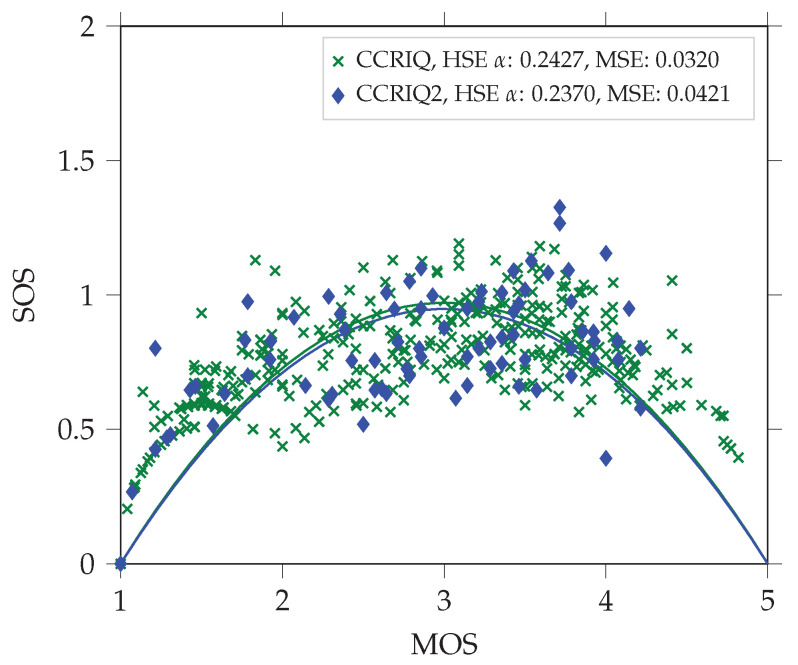
A comparison of HSE α parameters between the new CCRIQ2 and legacy CCRIQ data sets. We use the α parameters to fit the quadratic function proposed in [26]. It models the dependency between MOS and SOS. We analyze both data sets without unreliable subjects (screened according to [30] with the threshold correlation set to 0.6 for CCRIQ2 and 0.75 for CCRIQ).

We start the formal comparison with the HSE α parameter. CCRIQ scores result in an HSE α of 0.2427 (and MSE of the corresponding fit of 0.032). (Please keep in mind that we provide this value for only a subset of all CCRIQ scores. Namely, we utilize only the ratings for a High-Definition monitor.) CCRIQ2 scores correspond to HSE α equal to 0.237 (and MSE of the corresponding fit of 0.0421). Figure 6 shows the quadratic fits. Interestingly, this comparison presents CCRIQ2 as a data set of lower scores variability. We treat this result carefully since CCRIQ2 contains reliable scores from only 14 subjects. Although CCRIQ has a higher α, its 47 reliable subjects provide a better quadratic fit (represented by the lower MSE). This many subjects also means we can generally express greater confidence about information the results convey (just because of the larger sample from the subjects population). Summarizing our findings this far, we conclude the comparison of the HSE α suggests the two data sets are compatible.

Staying in line with Recommendation ITU-T P.913 [30], we use the two-sample unpaired Student’s *t*-test to check if CCRIQ and CCRIQ2 are different. Specifically, we check whether the same cameras are scored differently. For this, we use MOS scores of all images taken with a given camera. This generates one sample from CCRIQ and one sample from CCRIQ2. We then compare sample pairs for each camera (there are 23 cameras, and hence there are 23 pairs). Importantly, we only use scores of reliable subjects. (We filter out unreliable subjects using post-experimental screening of subjects. The method comes from Recommendation ITU-T P.913 [30]. We use a threshold correlation of 0.6 for CCRIQ2 and 0.75 for CCRIQ.) We apply the two-tailed test at the overall significance level of α = 5%. To compensate for multiple comparisons, we apply the most conservative, Bonferroni correction [32]. The correction results in testing each comparison with a stricter significance level of α/23, which is ≈0.0022. The tests show that no camera is scored significantly differently between the two data sets.

Since individual scores are ordinal data, we now compare CCRIQ and CCRIQ2 using the Mann–Whitney–Wilcoxon two-sample rank-sum test (also called Mann–Whitney *U* test). This test is recommended explicitly for comparing ordinal results. First, we group individual scores by a camera type. Then, we perform comparisons for pairs of groups, with each pair corresponding to a single camera type (there are 23 camera types, and hence there are 23 pairs). We apply the two-tailed test at the overall significance level of α = 5%. As previously, we use the Bonferroni correction (as a countermeasure for a multiple hypotheses testing problem). Some tests reject the null hypothesis that scores in both data sets come from populations with the same distribution. We see this outcome for 4 out of 23 cameras. Running a one-tailed test further shows that three (3) cameras receive higher scores in CCRIQ2 and one (1) camera scores higher in CCRIQ. Exact significant *p*-values are in Table 2. Differently put, the table shows only those cameras which are scored significantly differently in CCRIQ and CCRIQ2 data sets.

There is no unified trend explaining which cameras perform better in one data set or the other. For example, one cannot say that cameras with high pixel count perform better in CCRIQ. The analysis contradicts our expectation that low-resolution images will have similar scores in both data sets. Due to many factors having a possible influence on all scores, we are not able to make per-camera-type conclusions. The differences may come from content dissimilarity between the two subjective tests but may also be caused by the different testing environment. We conclude that, even when exploring new designs of subjective tests, care should be taken not to introduce too many factors that might have a potential influence on subjective scores.

The different outcomes of *t*-test and *U* test show the two cannot be used interchangeably. They test two different hypotheses. *t*-test tests a null hypothesis of equal means in two groups. On the other hand, *U* test tests a null hypothesis that the probability of randomly drawing an observation from one group that is larger or smaller than a randomly drawn observation from the other is equal to 0.5. In other words, it tests whether two groups have the same median.

Significant differences coming from the *U* test should be analyzed with caution. The authors of [9] point out that MOS scores may always be study-relative. Put another way, degradation conditions (here different cameras) may be similarly ordered in two tests, but absolute scores can still differ significantly. This does not mean the *U* test results are false. It means they should not be used as an ultimate measure of test dissimilarity.

Summarizing the results from this section, we conclude that CCRIQ and CCRIQ2 are compatible. Our findings do not present evidence for significant differences between the two. This suggests that both data sets can be combined. Such a combined data set should result in more accurate per-camera analyses. It also provides enough data points to train and test an NR metric.

## 4. Materials and Methods

We introduce the methods used grouped according to their use. We start by describing the subjective study performed. Then, we present the tools we used for the subjective scores integrity check.

### 4.1. Subjective Study Design

We carried out the subjective experiment at AGH University of Science and Technology, in Kraków, Poland. The experiment constituted a part of the teaching course “Multimedia Information Processing and Communications.” The course consisted of several multimedia topics, including subjective quality evaluation. The class lesson allowed us to conduct a 90-minute long experiment. However, students needed, on average, only 35 minutes to complete it.

**Figure 7:**
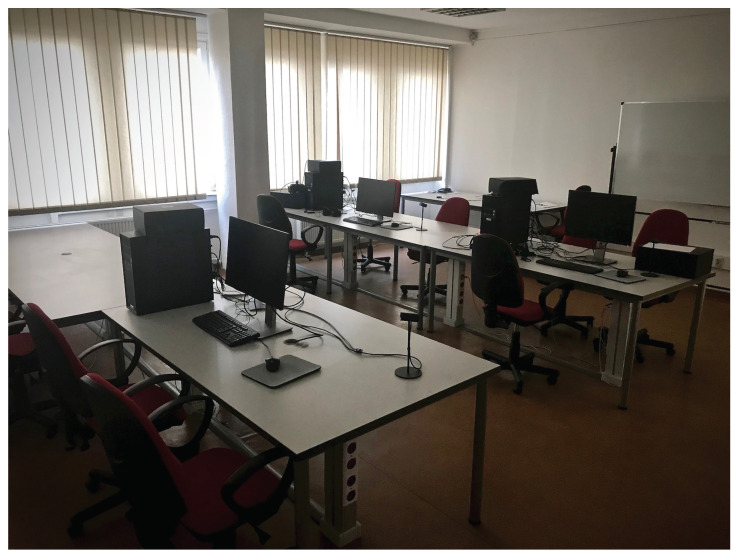
A photograph of the room next to the laboratory where we carried out the test. We show the neighboring room since the actual laboratory is not available at the time of writing this paper. The original laboratory has the same lighting conditions (concordant with Recommendation ITU-T P.913). This picture show very similar conditions.

The laboratory (see Figure 7) allowed for a controlled environment as specified by Recommendation ITU-T P.913 [30]. Illumination control was possible yet limited (to: “dark,” “dim,” and “bright”). We did not measure the precise illuminance level and color temperature of the lighting conditions. However, the study took place around sunset so stable artificial lighting was the main source of illumination. The subjects self-selected a comfortable viewing distance. Twenty-four students took part in the experiment at once. Hence, they could cross-watch their screens. The students used homogeneous Windows/Linux personal computers with 4+ gigabytes of random-access memory. They all had homogeneous Dell P1913 1440 × 900 (wide extended graphics array plus, WXGA+) screens. We did not measure the colorimetric properties of these displays.

As far as the demographics of the subjects is concerned, they were all “naïve” (“fresh”) viewers. They were all around 23–24 years old and students of AGH University. The subjects were 78% male and 22% female. All the subjects had a background in Information and Communication Technologies (ICT). Polish was their native language, but they also had command of English.

The experiment itself used the 5-level Absolute Category Rating (ACR) scale. We chose to use this scale as our goal was to provide training data for NR metrics. Since NR metrics should be referenceless by nature, we found the ACR method more appropriate than, for example, the pair comparison method [11] (p. 8). Differently put, we sought to provide training data for ACR-like NR IQA algorithms and hence using the ACR scale was the natural choice.

**Figure 8:**
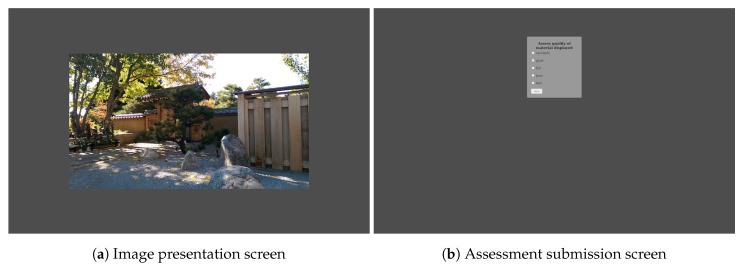
Two screens from our web-based software interface for conducting the subjective assessment.

Custom, web-based software (Figure 8) was used to present the content and to collect scores. The presentation order of images was randomized for each participant. Importantly, there was no time limit on displaying an image. Each study participant could watch the image for as long as necessary. In order to provide their rating, participants had to click the image. Only then was a separate screen with the rating scale displayed. Participants took, on average, approximately 10 s to watch and rate a single image.

Due to many subjects taking the experiment simultaneously, we were not able to control all of them at once. This resulted in non-identical subject pools in the training session and the two target sessions (VIME1 and CCRIQ2). Of the 24 subjects, 18 took part in the training session, 21 took part in the VIME1 session, and 19 took part in the CCRIQ2 session. This means some subjects participated in the VIME1 or CCRIQ2 session but had not been through the training session. This situation could have been avoided if a different subjective testing software has been used. The situation occurred because our testing interface had a limitation of having only one active session at a time for all study participants. Before switching from the training session to the next session, the one experimenter present in the laboratory asked whether all participants had finished the training. Everyone confirmed that they had, but the results presented a different picture.

The test started with a training session that consisted of 11 images (four from CCRIQ2 and seven from VIME1). After this, we presented the testers with 101 images (the VIME1 session). Again, after those, we showed them 88 images (the CCRIQ2 session). Importantly, the 11 images from the training session did appear in the VIME1 and CCRIQ2 sessions.

Although not perfect, we decided to use this experiment design since it had previously proven to be functional. Specifically, we were able to reproduce the results of selected classical subjective studies (i.e., those with visual content having artificially introduced distortions). For the sake of scientific correctness, we nevertheless underline the shortcomings of our design choices. No vision acuity or color blindness screening was performed. Furthermore, not all test participants took part in the training session. Finally, no control over the viewing distance was imposed and there was no precise control over lighting conditions and acoustic noise isolation. All those factors may have a confounding impact on our results.

### 4.2. Subjective Scores Integrity Check

We check the data integrity using three methods: (i) HSE α parameter analysis, (ii) comparing the MOS of each subject with the MOS of all subjects and (iii) applying the post-experimental screening of subjects. We choose to go beyond a simple comparison of MOS values as existing works [33,34] point to the weakness of this approach. Specifically, they highlight its inability to provide a comprehensive view of the judged quality.

We use the HSE α parameter as, according to the authors of [26], it both comprehensively summarizes results and checks their reliability. Furthermore, it also allows for checking comparability between different subjective studies. In short, the parameter describes the degree of variability in data (referred to as the standard deviation of opinion scores (SOS) by the authors). It does so across the whole range of the MOS scale (a continuous scale from 1 to 5 in our case). This is possible because of assumptions about no variability at scale extrema and highest variability in its middle (MOS equal to 3 in our case). The authors’ hypothesized quadratic relationship (described by a quadratic function) between variance and MOS reflects the two assumptions. The HSE α parameter expresses the exact shape of this relationship. Its value is a result of fitting the quadratic function into actual MOS–SOS pairs (one pair for each test material). To describe the accuracy of this fit, we follow the authors and report its mean squared error (MSE). As highlighted by the authors of [26], classical subjective tests of images or video streaming have an HSE α parameter with a value roughly between 0.01 and 0.22 (higher α corresponds to higher variability). We expect to observe similar or higher values for CCRIQ2 and VIME1 data sets. This potentially higher variability of our data sets may arise from the use of consumer content. A design using such content is in general less controlled than the classical approach (with artificially introduced distortions).

The second method we use compares each subject’s opinion with the general one (averaged over all subjects). To get a more reliable comparison, we aggregate individual opinion scores over camera type. In other words, we average opinion scores given by one subject to all images taken by a given camera type. It gives us the MOS for each subject (instead of less stable individual scores). Having a set of MOS scores for all camera types for a given subject, we compare it with MOS scores computed from opinion scores of all subjects. We end up with two vectors: (i) a personalized MOS score for each camera and (ii) a general MOS score for each camera. We then visualize the relation between the two using a scatter plot. Repeating the procedure, we get a set of scatter plots, one for each subject. If the data are consistent, we should see a positive linear relationship in most (or all) of the scatter plots.

A careful reader will notice more than 11 points in each scatter plot related to VIME1 (see Figure 5). This may seem unusual since we refer to camera type and there are 11 cameras in this data set. There are more data points in the scatter plots because we treat each post-processed version of an image as a separate camera type. In other words, the same image content with and without post processing constitutes two distinct camera types. This results in 26 camera types and thus 26 data points in each plot.

Importantly, we perform the scatter plot analysis not to compare the cameras, but to verify whether each study participant is following the general opinion trend. We specifically focus on the per-camera approach because it gives a sufficient number of data points. Differently put, there are enough points to visually see whether there is a correlation between personalized and global MOS. This analysis can be repeated by aggregating by other factors as well (e.g., by scene). However, we do not show scatter plots resulting from other aggregation strategies since none of them provide a sufficient number of data points.

Finally, we perform a post-experimental screening of subjects. Following Recommendation ITU-T P.913 [30], we check the correlation between the raw scores of each individual subject and the average raw scores of all subjects. We perform the computations on a per-image basis. Having the correlations for all subjects, we discard the worst outlier, then repeat the procedure. We treat as outliers subjects with a correlation below 0.75 (a value recommended for subjective tests of entertainment videos). We finish the procedure when there are no subjects with a correlation below 0.75. We expect to discard up to 10% of subjects.

## 5. Conclusions

With this paper, we hope to encourage the development of NR IQA algorithms for consumer content. We describe two new data sets, CCRIQ2 and VIME1, that provide consumer images and corresponding subjective scores. Those shall help train and test better NR metrics. We make CCRIQ2 and VIME1 available royalty-free through the CDVL [17] (search CDVL using the keyword “ccriq2+vime1”).

We also check data integrity of the two data sets (thus addressing hypothesis #1). We show that scores from the VIME1 data set are inconsistent. This represents a case for which the classical subjective test design fails to work. The situation is unusual since evaluations of the similarly designed CCRIQ2 provide consistent results. This suggests a series of questions. Maybe the VIME1 design is too close to consumer camera usage? Where then is the borderline beyond which the classical subjective study design ceases to work? Can we think of developing NR metrics if we do not know how to design subjective studies that correctly generate ground truth data? We put forward these questions as they are crucial for advancing consumer content evaluation and NR metrics research.

Our work may as well be used as a benchmark for data integrity checking tools (thus addressing hypothesis #2). Let us boil this down to the following recommendations. The results indicate that the HSE α parameter does not seem to be a reliable mechanism for comparing subjective studies. In particular, it should not be used as an ultimate measure of similarity. Even though two studies may have similar HSE α parameters, one may be consistent, and the other may not be. We recommend investigating the distribution of scores through scatter plots to alleviate this problem. The plots should show each tester’s opinion juxtaposed with the general opinion among all testers (for details, see Section 4.2). The apparent correlation between testers suggests data integrity. To further verify this claim, we advocate using the post-experimental screening of subjects (as defined in Recommendation ITU-T P.913 [30]).

Our last contribution is the comparison of CCRIQ2 to its legacy counterpart, CCRIQ. The qualitative and quantitative analyses indicate that the two data sets are compatible. This means they can potentially be combined. Such a combined, more extensive data set should allow for performing more trustworthy per-camera inferences. It would also constitute good training data for NR metrics. We leave exploring the benefits of the combined data set for future research.

Similarly, we let explaining VIME1 inconsistency be another future goal. Among the potential causes for the lack of integrity are mixing of vertically and horizontally aligned images, inclusion of post-processed images, utilizing a narrow range of contents, allowing many photographers to capture the same scene, substantially redefining the meaning of content degradation condition (HRC), and, finally, performing the study as an element of a teaching course. At this point, we theorize that the mixing of vertical and horizontal images may be the most probable reason for the inconsistency.

## Figures and Tables

**Table 1:** A comprehensive comparison of the new CCRIQ2 + VIME1 data set with the similar, existing data sets. We choose to include in the comparison only the data sets that contain user-generated content. This is to assure our comparison addresses only the data sets targeting NR metrics development. The *mod. ACR* label means a modified version of the classical 5-level ACR method [11] (p. 6) is used in a data set. C+V stands for CCRIQ2 + VIME1, LIVE ItW for Live In the Wild Image Quality Challenge, LIVE QM for LIVE-Qualcomm Mobile In-Capture, KonIQ for KonIQ-10k Image, KoNViD for KoNViD-1k Video, CID for CID2013 Camera Image Database and CQ for CCRIQ. cs stands for crowdsourcing and n/a for not available.

Data Set	CID	CQ	LIVE ItW	LIVE QM	KonIQ	KoNViD	ITS4S	ITS4S2	ITS4S3	C+V
**Year**	2013	2014	2016	2017	2017	2017	2018	2019	2019	2020
**Data Type**	image	image	image	video	image	video ${}^{1}$	video	image	video	image
**No. of Stimuli**	480	392	1162	208	10,073	1200	813	1473	594	189
**Avg. No. of Ratings per Stim.**	31	26, 27, or 53	175	39	120	>50	33	16	15	19
**No. of Unique Scenes**	8	18	1162	54	10,073	1200	813	1473	594	11
**Total No. of Subjects**	188	53	>8100	39	1467	642	51	16	87	24
**Scoring Method**	mod. ACR	ACR	mod. ACR	mod. ACR ${}^{2}$	ACR	ACR	mod. ACR	ACR	mod. ACR	ACR
**Content Res. [MP]**	1.92	2.073 and 8.294	0.25	2.074	0.786	0.518	0.922	0.135 to 233.293	0.307 to 2.073	0.922 to 19.962
**No. of Devices**	79	23	>15	8	1265	n/a	n/a	>240	∼15	34
**Env.**	lab	lab	cs	lab	cs	cs	lab	lab	public venue	lab-like

${}^{1}$ The only video data set that contains audio. It is present in 97% of stimuli. ${}^{2}$ One more, custom scoring method is utilised. It is referred to in [20] as “distortion-guided”.

**Table 2:** Significant *p*-values of the two-sided (fourth column) and the one-sided (fifth and sixth columns) *U* tests. The tests compare the new CCRIQ2 and legacy CCRIQ data sets. We group individual scores by a camera type. Then, we apply *U* test for pairs of groups (one group of scores in the pair comes from CCRIQ and one from CCRIQ2). The third column presents sensor sizes of each camera. We show them to verify the hypothesis of low resolution images being similarly scored in both data sets.

Camera Type	Camera Label	Sensor Size [Million Pixels]	*p*-value		
			CCRIQ ≠ CCRIQ2	CCRIQ < CCRIQ2	CCRIQ > CCRIQ2
smartphone	H	5	0.000631	0.000316	
smartphone	I	5	0.000336		0.000168
DSLR	K	8	0.000827	0.000413	
smartphone	R	13	0.000132	0.000659	

## Supplementary Materials

The following is available online at https://www.mdpi.com/2313-433X/6/3/7/s1. Spreadsheet S1: ccriq2_vime1_tidy.csv contains 3745 opinion scores for images from the VIME1 and CCRIQ2 data sets. Each row corresponds to a single opinion score of a single study participant. The CSV file has 10 columns: (i) *lab*—The location the subjective study has been performed (i.e., AGH UST); (ii) *Tester_id*—A unique identification number of each study participant; (iii) *Exp*—A name of a test session (either “vime1” or “ccriq2”); (iv) *PVS*—A name of an image presented to a study participant; (v) *Scene*—A scene, of which a photograph constitutes a test material (a.k.a. PVS); (vi) *Camera*—An identification string of a camera used to capture the image (a.k.a. PVS or test material); (vii) *OS*—An opinion score of the study participant expressing his/her judgment of the overall image quality; (viii) *Timestamp*—A timestamp of the opinion score submission; (ix) *Camera_w_ver*—A string combining the camera identifier with a version of the test material (versions other than “ver1” mean the image is post-processed); (x) *Orientation*—The test image orientation (horizontal or vertical; applicable only to the VIME 1 session).
